# The relationship between compression garments and electrocardiogram signals during exercise and recovery phase

**DOI:** 10.1186/s12938-019-0645-2

**Published:** 2019-03-19

**Authors:** Lan Thi Nhu Nguyen, David Eager, Hung Nguyen

**Affiliations:** 1 School of Biomedical Engineering, University of Technology Sydney, Broadway, NSW Australia; 2 Faculty of Engineering and IT, University of Technology Sydney, Broadway, NSW 2007 Australia; 3 Faculty of Science, Engineering and Technology, Swinburne University of Technology, Hawthorn, VIC 3122 Australia

**Keywords:** Cardiovascular, Compression garments, QTc, ECG, Heart rate, Sport

## Abstract

**Background:**

The direction of the current research was to investigate whether electrocardiogram (ECG) signals have been impacted by using compression garments during exercise and recovery phase. Each subject is non-athletes, conducted two running tests, wearing either non-compression garments (NCGs) or compression garments (CGs) throughout experiments and 2-h of the recovery phase. Experiment 1 (number of participants (n) = 8; 61.4 ± 13.7 kg, 25.1 ± 3.8 years, 165.9 ± 8.3 cm) focused on the exercising phase while Experiment 2 (n = 14; 60.9 ± 12.0 kg, 24.7 ± 4.5 years, 166.0 ± 7.6 cm) concentrated on the recovery phase. Electrocardiogram (ECG) data were collected through wearable biosensors.

**Results:**

The results demonstrated a significant difference between compression garments and non-compression garments at the end of the tests and from 90 min onwards during the recovery phase (p < 0.05). Corrected QT (QTc), ST interval and heart rate (HR) indicated the significant difference between NCGs and CGs. Conclusion: Based on the findings, the utilization of compression garments showed a positive influence in non-athletes based on the quicker recovery in HR, ST, and QTc.

## Introduction

Compression garments are becoming popular for sports activities in athletes and non-athletes. Many studies have claimed that compression garments may have positive effects and could increase performance. For example, during distance running, wearing CGs correlates with significantly lower muscle activation and longer median frequencies which might prevent muscle-fatigue and enhance running performance [1]. Other research investigating the use of upper body CGs indicates that performance and comfort can be increased as a result of enhanced proprioceptive cues during upper-body movements in high-level athletes [2]. Similarly, there was an improvement in performance resulting from altering the running technique and reducing perceived exertion [3]. However, another article has also identified non-affective major hemodynamic parameters, sensation, physiological factors and performance compared with loose-fit breeches [4]. Essentially, the effects of compression garments during exercise remain incomplete.

Compression garments have also been recommended as a recovery tool due to many advantages. For instance, CGs decreased muscle soreness post 24 h of 25 repetitions on a modified preacher curl machine exercise [5], post 48 h of three treadmill runs at a 6% elevation rate [6] or after 72 h of a vertical jump test [7]. CGs also represented a positive effect on delayed onset muscle fatigue and swelling [8]. While the studies as mentioned above showed the usefulness of CGs on recovery, there are still many other studies which indicated that the extent of the recovery rate or peripheral fatigue following activity was not affected by CGs [9]. Therefore, the effects of CGs on recovery need further investigation.

Overall, many studies have shown that a cardiovascular function has some correlation to exercising performance and recovery. Substantial evidence exists from previous research demonstrates that the increase in heart rate variability (HRV) indicates lowering risk profiles and a higher number of risk factors related to the decrease of HRV [10]. Additionally, heart rate (HR) post-exercise can be an effective parameter in designing the training strategy of both non-athletes and athletes and plays an important role in performance’s monitoring [11, 12]. For instance, slower heart rate recovery (HRR) correlated to the higher risk of autonomic dysfunction endothelial dysfunction diabetes, cardiovascular mortality, and metabolic syndrome [13].

The increase of hypertension also relates to higher HR [14]. Moreover, a higher value of resting HR can predict the risk of increasing hypertension [15]. However, dynamic mechanisms of HR underlying the influence of compression garments on wearers are not well understood [16]. In this study, effects of CGs were investigated and focused on non-athletes only.

Additional parameters comprised ST, QRS, TpTe, QT and corrected of QT (QTc) were provided via ECG signals. Many previous investigations identified the strong relationship between the improvement in cardiac arrhythmias and prolongation of QTc or QT intervals [17]. An adverse effect on the performance in training of athletes has associated with the higher values of QT and QTc dispersion [18]. Similarly, research of Mandyam (2012) suggested that the development of incident atrial fibrillation and stroke were also affected by a longer QT interval [19]. Moreover, there are many studies associated with ST-elevation such as a relationship between ST-elevation and predicting left ventricular [20]. However, the ST intervals are still lacking investigation. To the best of our knowledge, although some studies relating to a monitoring system and an automatic wearable ECG classification were published [21], there is no research related to the effects of using CGs on ECG signals on both during and post-exercise.

This present study examines the underlying mechanism of cardiovascular effects associated with compression garments through exercise and recovery based on ECG signals.

## Methods

### Participants

Eight healthy volunteers [age: 25.1 ± 3.8-year-old, weight: 61.4 ± 13.7 kg (kg), height: 165.9 ± 8.3 cm (cm)], including three women and five men, attended for Experiment 1. Fourteen healthy subjects (age: 24.7 ± 4.5-year-old, height: 166.0 ± 7.6 cm, weight: 60.9 ± 12.0 kg), including seven women and seven men, conducted in Experiment 2.

All participants did not have a smoking history and were non-athletes. Exclusion criteria comprise respiratory, cerebrovascular and cardiovascular disease. The consumption of alcohol and caffeine were not allowed for 24 h before the trials. The volunteers also were required to have a healthy night sleep. All subjects finished consent forms and the questionnaire of a basic medical situation before the experiments. The protocols were accepted by The Human Ethics Committee at the University of Technology Sydney (UTS). (Study 1: 2014000844; Study 2: ETH16-0696). The detailed parameters of the subjects of the two studies are indicated in Table 1.

**Table 1:** Subjects characteristics

Parameters	Study 1 (mean ± std)		Study 2 (mean ± std)	
	Men N = 5	Women N = 3	Men N = 7	Women N = 7
Age (years)	26.2 ± 3.1	23.3 ± 4.9	25.8 ± 4.9	23.7 ± 40
Height (cm)	170.6 ± 5.5	158.0 ± 5.6	171.6 ± 5.0	160.4 ± 5.1
Weight (kg)	70.6 ± 6.0	46.0 ± 3.6	70.7 ± 6.6	51.1 ± 6.8
BMI (kg m^− 2^)	24.4 ± 3.4	18.4 ± 0.9	24.1 ± 2.6	19.8 ± 1.9

### Test conditions and compression garments

**Fig. 1:**
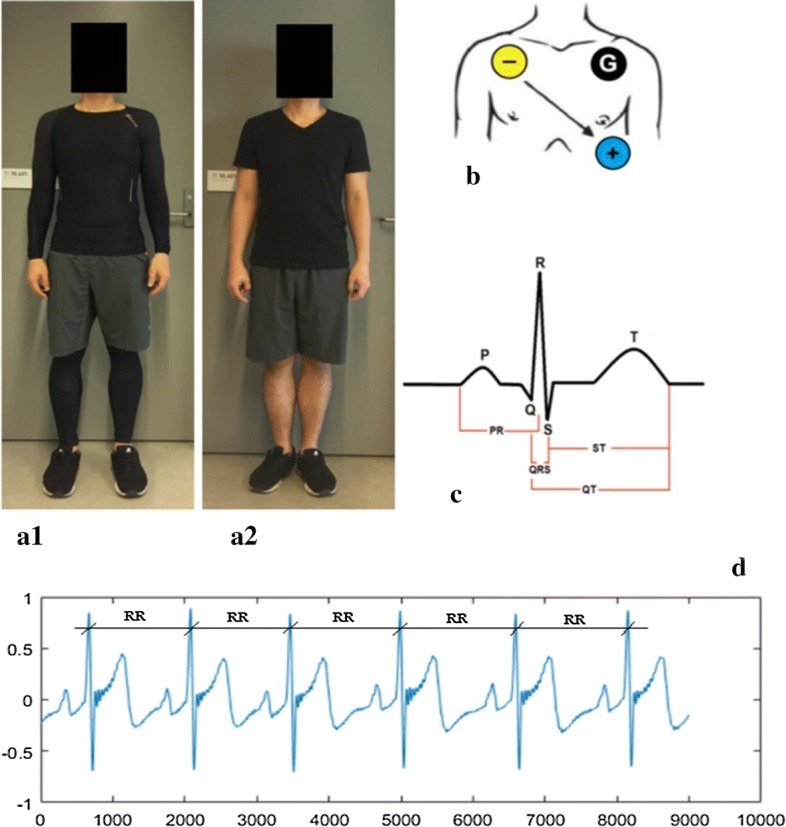
Subject use CGs (**a1**) and NCGs (**a2**). Position of lead II (**b**). Intervals detection (**c**). RR intervals (**d**)

Compression garments with the whole-body were used in the experiments. SportSkins Classic CGs come from Skins, Campbelltown, New South Wales, Australia. The compression garments comprised a long-sleeved top (neck–waist) and full-leg pants (waist–ankle). These components included 24% of Roica Spandex and 76% of Meryl Microfiber and Nylon. Based on participants’ body stature and mass, the corrected size garments were chosen for CGs according to the manufacturer’s suggestion. The NCGs were short and T-shirt which provided 0% level of pressure during the experiments. The volunteers used the same shoes and socks in both tests. The types of garments are shown in Fig. 1.

Each subject came to the laboratory two separate days throughout the experiments period. Two kinds of garments (NCGs and CGs) were used randomly through two exercises on a treadmill. The normal range of temperature was set from 20 to 22 °C in both testing days.

### Experimental design

On two different days, subjects conducted in two running trials with compression garments and without compression garments. Before the experiments, participants attended a rest of about 10 min. ECG of rest-data was calculated during this step from Thought Technology Ltd, Canada using a Flexcomp Infiniti Monitor. ECG electrodes were applied following the position of lead II (Fig. 1).

Experiment 1: Subjects conducted a 10-min running on 0% grade at six km/h of a treadmill. Following 2 min of running, volunteers were requested for data collection about 1.5 min (90 s) by stopping on the treadmill. Subsequently, the velocity was increased by 1 km/h following each pre-determined speed. The protocol continued when participants reached a velocity of 11 km/h. The data were collected as soon as subjects completed each speed during the stop.

Experiment 2: Subjects conducted a 10-min running on 0% grade at 6 km/h of a treadmill. Following every 2 min of running, the treadmill was speeded up by 1 km/h. The tests remained until the velocity of 11 km/h was finished. After that, participants took 2 h rest for recovery. ECG data were collected during the 2 h following the tests.

### Data collection

**Fig. 2:**
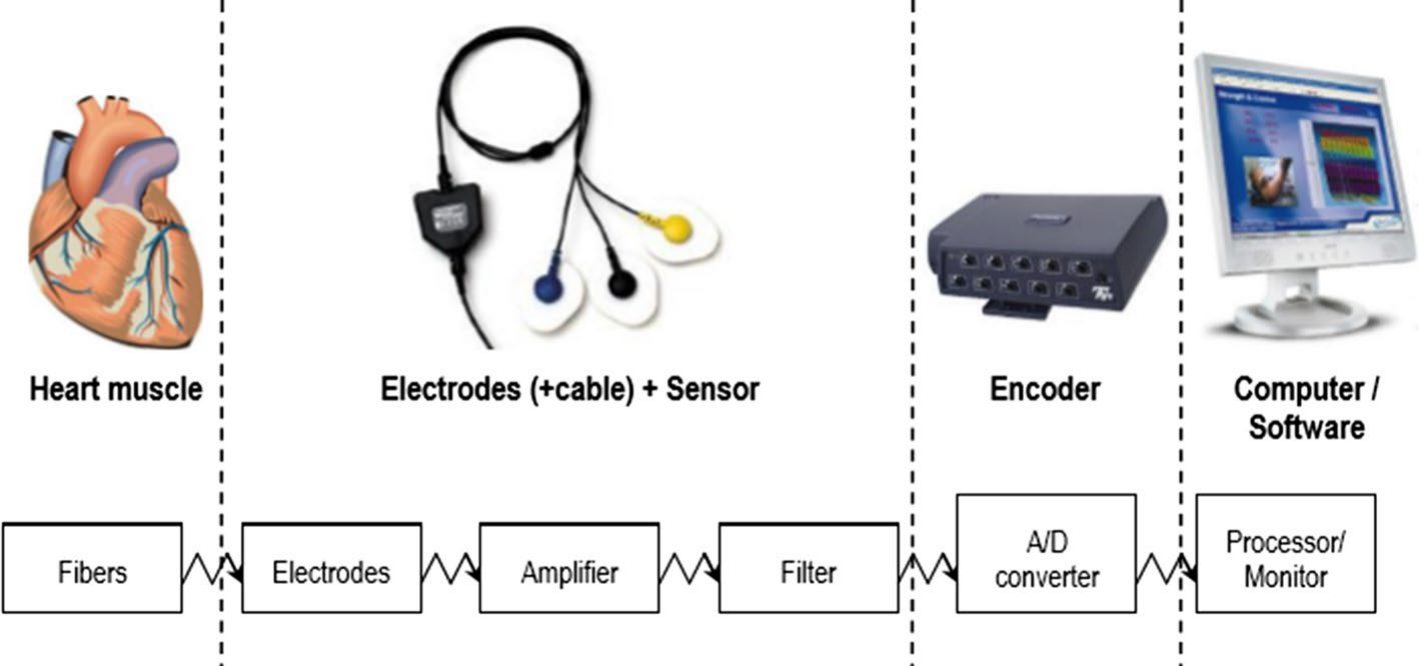
ECG detection

The electrical signals, caused by heart muscle, were detected by the ECG sensor. The electrodes captured this electrical signal. After that, the sensor amplified and filtered the signal. Digital signals were then converted by the encoder. Computer processed, recorded and displayed this digital signal. The BioGraph Infiniti software was used for this step. All the progress was showed in Fig. 2.

The sample rate is at 2048 samples per second. The ECG amplifier is the ECG-Flex/Pro (SA9306M). Heartbeat was detected by collecting the electrical activity of the heart. The measurement’s unit is in millivolts (1 µV).

Notch filters were applied to avoid line interference noise. The power line frequency was removed by the ECG notch filter (60 or 50 Hertz). The distance of 3 meters (10 feet) from any radio transmitting devices and 1 m (3 feet) from any electronic equipment was required for the device.

Matlab was applied for statistical analysis and calculating peaks. Mean values (mean) and standard deviation (std) were assessed for all statistical results. The significant level was chosen when the value of p was lower than 0.05. All data analyses were conducted according to a version of Matlab 2016b.

HRV parameters were calculated throughout the 90 s data collection period. The intervals were assessed during the first 10 s immediate stop for analysis in Experiment 1.

The values are represented by an assessment of RR interval variability. The RR interval was calculated as the interval from the peak of one QRS complex to the peak for the next, shown in Fig. 2. HR was calculated from RR intervals (second), following Formula (1). ECG intervals including ST, TpTe, QRS, QT intervals were assessed from positions of Tp, Te, S, Q during 20 s following each recovery phase of 10-min in Experiment 2. Although many various formulas can calculate QTc, the Bazett was recommended as the most popular description to reveal the considerable findings. The mentioned calculation is represented in Formula (2).

1 $$ HR = \frac{60}{RR} $$

2 $$ QTc = \frac{QT}{{\sqrt[{}]{RR}}} $$

## Results

### Heart rate variability

**Fig. 3:**
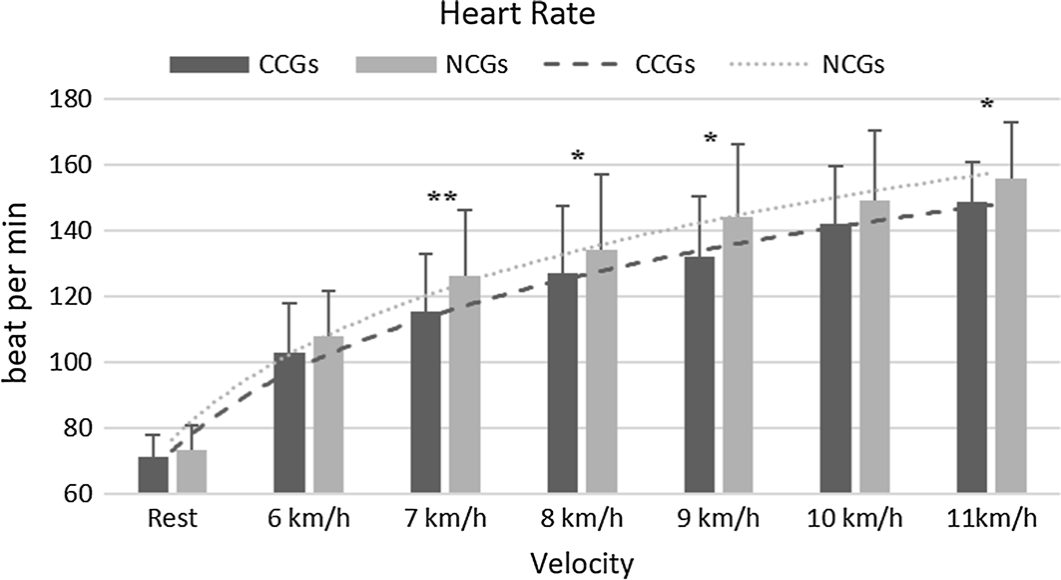
Heart rate during exercise in both groups of CGs and NCGs.*Significant difference (*p < 0.05, **p < 0.01)

Experiment 1, HRV parameters were considered within 90 s of the collection period. The results demonstrated some statically significant differences between using CGs and NCGs in HR after completing velocity of 7 km h^−1^ (NCGs: 126.2 ± 19.9, CGs: 115.2 ± 17.5, p = 0.0033), of 8 km h^−1^ (NCGs: 134.0 ± 23.2, CGs: 127.2 ± 20.2, p = 0.0473), of 9 km h^−1^ (NCGs: 144.0 ± 22.2, CGs: 132.1 ± 18.3, p = 0.0251), of 10 km h^−1^ (NCGs: 149.2 ± 21.4, CGs: 141.9 ± 17.9, p = 0.0804) and of 11 km h^−1^ (NCGs: 155.9 ± 17.1, CGs: 148.6 ± 12.4, p = 0.0165), following Fig. 3. Other parameters demonstrated a non-significant difference between the two groups of garments.

**Fig. 4:**
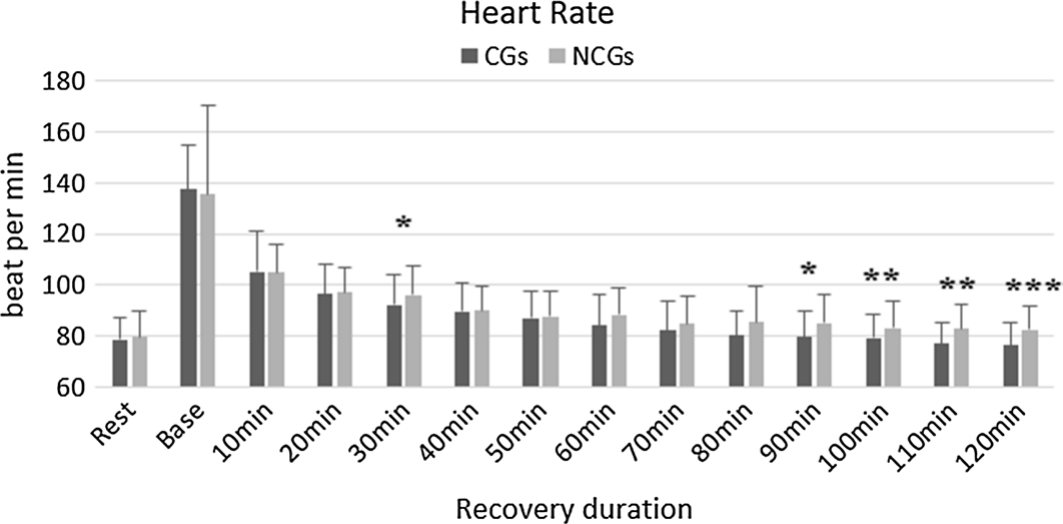
Heart rate in both groups of CGs and NCGs post-exercise.*Significant difference (*p < 0.05, **p < 0.01, ***p < 0.001)

Experiment 2, CGs group indicated significantly lower HR compared with NCGs group after recovery of 30-min (CGs: 92.1 ± 11.8, NCGs: 96.0 ± 11.5, p = 0.0485). Similarly, the significant influence of the use of CGs was revealed from the recovery of 80 min onwards. For instance, the results were demonstrated to have significant differences at the recovery of 80-min (NCGs: 85.4 ± 13.9, CGs: 80.1 ± 9.6, p = 0.0376), at the recovery of 90-min (NCGs: 85.2 ± 10.8, CGs: 79.5 ± 10.5, p = 0.0066), at the recovery of 100-min (NCGs: 83.1 ± 10.5, CGs: 79.1 ± 9.6, p = 0.0072), at the recovery of 110-min (NCGs: 82.8 ± 9.5, CGs: 76.9 ± 8.3, p = 0.001) and at the recovery of 120-min (NCGs: 82.3 ± 9.2, CGs: 76.4 ± 8.5, p = 0.0001). There was no significant change between using CGs and using non-CGs in other HRV values. The results are shown in Fig. 4.

### Intervals

**Fig. 5:**
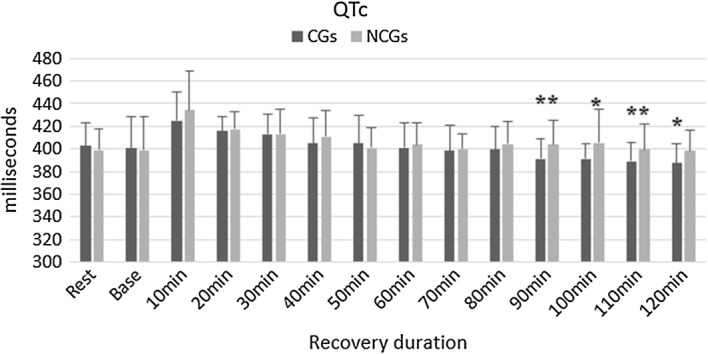
QTc in both groups of CGs and NCGs post-exercise.*Significant difference (*p < 0.05, **p < 0.01)

Experiment 2, the parameters concluded a significantly lower corrected QT intervals in the group using CGs at 90-min recovery (NCGs: 403.8 ± 21.6 ms, CGs: 391.3 ± 18.0 ms, p = 0.0035). The QTc was also influenced by the utilization of CGs with p = 0.0369 (NCGs: 405.3 ± 29.8 ms, CGs: 391.0 ± 13.1 ms) at 100-min recovery. Consistent with this, the value was affected by using CGs at the recovery of 110-min (NCGs: 399.1 ± 22.5 ms, CGs: 388.9 ± 16.5 ms, p = 0.0073). At the recovery of 120-min, there was a significant difference between two groups of garments (NCGs: 398.3 ± 18.1 ms, CGs: 387.6 ± 16.7 ms, p = 0.0128). The findings are shown in Fig. 5.

**Fig. 6:**
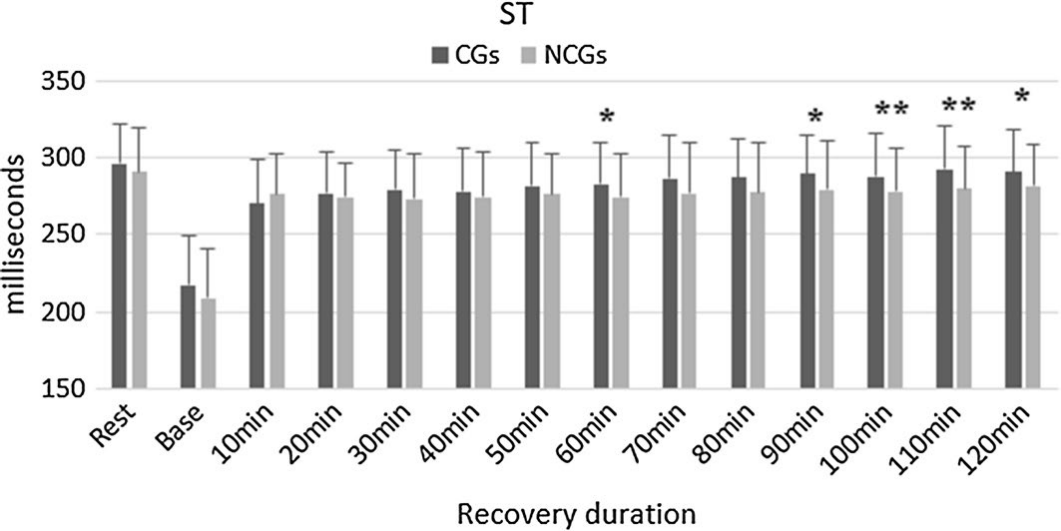
ST in both groups of CGs and NCGs post-exercise.*Significant difference (*p < 0.05, **p < 0.01)

After 60-min recovery, ST intervals reported a significant difference with p = 0.0269 (NCGs: 274.3 ± 28.1 ms, CGs: 282.6 ± 26.6 ms). Additionally, the application of CGs at 90-min recovery also determined a substantial difference in p = 0.0452 compared with NCGs with CGs: 289.2 ± 25.5 ms and NCGs: 279.4 ± 30.9 ms. At the recovery time after 100 min and 110 min, the finding demonstrated p < 0.001 with NCGs: 278.2 ± 28.3, CGs: 287.5 ± 28.0 ms (p = 0.0008) and NCGs: 279.5 ± 27.6 ms, CGs: 292.2 ± 28.1 ms (p = 0.0005), respectively. After 120 min of the recovering phase, the results reported a significant change in ST intervals between NCGs: 281.7 ± 27.2 ms and CGs: 290.8 ± 27.2 ms with p = 0.0216. The findings are shown in Fig. 6.

## Discussion

At the end of the running protocol, Experiment 1 indicated a significantly lower HR (p < 0.05). The present result is consistent with previous studies. For instance, in a study of well-trained runners, at a low intensity from 8 to 10 km/h, HR showed no significant difference between CGs and NCGs. However, at the high intensity from 12 to 18 km/h, HR showed a significantly lower value in CGs group than NCGs group [22]. In the current research, the significant difference in HR was found at a velocity of 7 km/h afterward. The level of intensity was lower than the abovementioned study because all volunteers are non-trained runners (non-athletes). Based on the observation, the significantly lower heart rate could be found when participants reached the high intensity and were exhausted. Consistent with this, earlier research of incremental cycling bouts demonstrated a significantly lower value of HR when wearing CGs compared with the application of regular above-knee cycling shorts [23].

Based on the results of higher performance in a study of an archery-competition, the lower HR may have a relationship to lower stress and more concentration [16]. This finding is also consistent with research involving ten golfers and eleven pitchers relating to the enhancement of performance in driving accuracy, fastball accuracy, chipping accuracy and shot accuracy by the use of CGs [24].

In Experiment 2, both NCGs and CGs indicated a decrease in HR throughout the recovering time. Through the first 80-min recovery, the application of CGs demonstrated non-significant difference compared with NCGs. This investigation is consistent with some previous research which reported no alteration between CGs and NCGs in heart rate of 6 steps incremental experiment after 20-min recovery on a kayak ergometer [25] and 3 running power trials of post 20-min intermittent maximal anaerobic [26]. Similarly, research of 40 km cycling time trial performance [27] and a match simulation exercise [28] determined a non-significant difference after half-hour recovery in HR in a comparison between wearing lower-body CGs (full long-leg pants) and using NCGs. Additionally, after 60-min recovery of a maximal cycling ergometer protocol, the findings also suggested no effects by using compression stocking compared with NCGs [23]. However, the results showed the significant lower HR (p < 0.05) after 80-min recovery in the participants with compression clothing compared with without compression garments.

Enhancement of redistribution of blood flow and blood flow was associated with a lower mean heart rate [24]. Additionally, glycogen depletion can be caused by high-intensity exercise relating to inadequate muscle glycogen synthesis. However, the issue can be solved by the development of blood flow via the utilization of CGs [27]. The quicker recovery of HR after the recovery of 80-min using CGs supports many previous studies associated with the suggestion of CGs as a recovering strategy [29], [30]. This investigation of the current research is that compression garments may have benefits after the running exercise in HR.

The present study investigated a significant lower QTc when using compression garments compared with non-compression garments after a 90-min of recovering time (p < 0.05). Similarly, an earlier study indicated a shorter value of QTc at the end of a running experiment in CGs group [31]. The previous finding proved that the longer QTc related to the development of atrial fibrillation and incidence of stroke [19]. Moreover, a significant increase in cardiac arrhythmias also associated with the prolongation of QTc [18]. The current results are consistent with our previous observation suggesting that the pressure of CGs may effect on QTc parameters [31]. Based on the findings of lower QTc, CGs may have a positive influence on the recovering phase.

In this present study, ST intervals indicated the decrease at the end of the tests and the increase of normal parameters throughout the recovering time. The application of CGs showed a higher value of ST intervals during the recovery. At 60-min of recovering time and after 90-min recovery, CGs demonstrated significantly longer ST intervals compared with NCGs (p < 0.05). Specifically, participants wearing CGs reached the normal parameters of ST intervals quicker than using non-CGs. Evidence of previous findings concluded that ST intervals correlated with low diastolic blood pressure (DBP) and high systolic blood pressure (SBP) [32]. The observation of the current result supported these previous results associated with the influence of CGs and indicated significantly higher SBP parameters during the standing phases and the supine [33].

## Conclusion

Based on the mentioned positive influence of cardiovascular function, the application of whole body compression garments may support both exercise and recovery phase. The current findings included a quicker recovery in heart rate, ST, and QTc. Between activity episodes, compression garments should be suggested to athletes and coaches for improving the recovery. The above observations can increase the training’s quality and lead to enhancement in subsequent performance. Further observations are required to investigate the underlying mechanisms of compression garments on recovery strategy and performance in athletes.
